# Let-7a regulates EV secretion and mitochondrial oxidative phosphorylation by targeting SNAP23 in colorectal cancer

**DOI:** 10.1186/s13046-020-01813-6

**Published:** 2021-01-14

**Authors:** You Dong Liu, Xiao Peng Zhuang, Dong Lan Cai, Can Cao, Qi Sheng Gu, Xue Ni Liu, Bin Bin Zheng, Bing Jie Guan, Liang Yu, Ji Kun Li, Hui Bin Ding, Dong Wang Yan

**Affiliations:** 1 Department of General Surgery, Shanghai General Hospital, Shanghai Jiao Tong University School of Medicine, Shanghai, 200080 China; 2 Department of Ophthalmology, Shanghai General Hospital, Shanghai Jiao Tong University School of Medicine, Shanghai, 200080 China; 3 Department of General Surgery, The People’s Hospital of Rugao, Rugao, Jiangsu 226500 China

**Keywords:** Let-7a, SNAP23, Colorectal Cancer, Extracellular vesicles, Oxidative phosphorylation

## Abstract

**Background:**

MicroRNAs (miRNAs) are abundant in tumor-derived extracellular vesicles (EVs) and the functions of extracellular miRNA to recipient cells have been extensively studied with tumorigenesis. However, the role of miRNA in EV secretion from cancer cells remains unknown.

**Methods:**

qPCR and bioinformatics analysis were applied for determining extracellular let-7a expression from CRC patient serum and cells. Nanosight particle tracking analysis was performed for investigating the effect of let-7a on EV secretion. Luciferase reporter assays was used for identifying targeted genes synaptosome-associated protein 23 (SNAP23). In vitro and in vivo assays were used for exploring the function of let-7a/SNAP23 axis in CRC progression. Bioenergetic assays were performed for investigating the role of let-7a/SNAP23 in cellular metabolic reprogramming.

**Results:**

let-7a miRNA was elevated in serum EVs from CRC patients and was enriched in CRC cell-derived EVs. We determined that let-7a could suppress EV secretion directly targeting SNAP23. In turn, SNAP23 promotes EV secretion of let-7a to downregulate the intracellular let-7a expression. In addition, we found a novel mechanism of let-7a/SNAP23 axis by regulating mitochondrial oxidative phosphorylation (OXPHOS) through Lin28a/SDHA signaling pathway.

**Conclusions:**

Let-7a plays an essential role in not only inhibiting EV secretion, but also suppressing OXPHOS through SNAP23, resulting in the suppression of CRC progression, suggesting that let-7a/SNAP23 axis could provide not only effective tumor biomarkers but also novel targets for tumor therapeutic strategies.

**Supplementary Information:**

The online version contains supplementary material available at 10.1186/s13046-020-01813-6.

## Background

Colorectal cancer (CRC) incidence ranks third in the world affecting over 1 million new cases per year [1]. Early diagnosis and effective treatment such as endoscopic resection have improved the survival of patients, but the prognosis of advanced CRC patients remains poor. Genetic and microenvironment factors have been identified as critical factors for tumors, causing the complex lethality and heterogeneity of CRC [2, 3]. However, the discovery of effective biomarkers and molecular targets for therapy are crucial.

Extracellular vesicles (EVs) are microvesicles derived from different types of cells and play critical roles in cellular communication by transporting information cargo, including proteins, mRNAs and microRNAs (miRNAs) [4]. Numerous researches have reported that tumor-derived EVs, which are enriched in the body fluids of cancer patients, promoted tumor angiogenesis, metastasis or chemotherapy resistance in CRC [5–8].

miRNAs are small noncoding RNAs that post-transcriptionally regulate gene expression. Although miRNAs are closely associated with cancer development, there is a limited understanding of their multiple mechanisms in tumor-derived EVs. Previously, the functions of extracellular miRNA to recipient cells have been extensively studied that normal fibroblasts or macrophages could be activated to cancer-associated cells by tumor-derived extracellular miRNA, further promoting tumor progression in different molecular pathways [9, 10]. These pathways also include an emerging hallmark of malignancy “metabolic reprogramming”. For examples, Yan et al. uncovered that extracellular miR-105 derived from cancer cells mediated metabolic reprogramming of stromal cells to promote tumor growth [11].

However, the regulatory EV secretion net-work in donor cells such as cancer and cancer-associated cells remains obscure. Mounting evidence suggests that the suppression of cancer-derived EVs could be a novel method for tumor therapy [12]. Recent studies have reported that miRNA could regulate EV secretion to keep itself steady-state level. For instance, miR-26a has been found to be related to EV secretion, resulting in the inhibition of prostate cancer progression [13]. Besides, the relationship between EV secretion and reprogrammed metabolism in cancer cells has not been identified before.

In our study, we demonstrated that let-7a miRNA, as a classical tumor suppressor, let-7a miRNA was upregulated in serum EVs from CRC patients and was enriched in CRC cell-derived EVs. We hypothesized that let-7a could regulate EV secretion in CRC. We identified the effect of let-7a on EV secretion, which downregulated the intracellular levels of let-7a via directly targeting SNAP23, a controller of the docking and release of multi-vesicular bodies (MVBs). Furthermore, we found a novel mechanism of let-7a, as an important regulator of cancer cell metabolism [14], by regulating mitochondrial oxidative phosphorylation (OXPHOS) that are involved in EV secretion. Thus, we show that let-7a plays an essential role in not only inhibiting EV secretion, but also suppressing OXPHOS by targeting SNAP23.

## Methods

### Cell lines and clinical tissue specimens

The human CRC cell lines SW48, SW480, HT29, SW620 and normal colon epithelial cell line FHC were grown in DMEM supplemented with 10% fetal calf serum (FCS). After the thawing of the parental stock, all the cells were passaged within 6 months. Human serum and tissue specimens were collocated from CRC patients and healthy controls (HC) in Shanghai General Hospital. All experimental procedures were approved by the Ethics Committee of Shanghai General Hospital.

### EV isolation and identification

EVs from the culture medium were isolated by a differential ultracentrifugation (UC) [13, 15]. Cells were transplanted into 10 cm plates and changed DMEM with 10% EV-depleted FCS. After 48 h, the culture medium was collected and centrifuged at 2000×g for 15 min to remove contaminating cells. Subsequently, the supernatant was filtrated through 0.22 μm filter (Millipore, Billeria, MA). Then, the medium was centrifuged for 120 min at 120,000×g to pellet the enriched EVs (Optima L-100XP, Beckman, USA). The pelleted EVs were washed with PBS and centrifuged at 120,000 for another 120 min. For miRNA detection in serum samples, EVs were isolated from serum samples using UC or Exoquick™ Reagent precipitation (System Biosciences, Mountain View) according to these methods [16, 17]. Briefly, 750 μl of serum was diluted with phosphate-buffered saline (PBS) and centrifuged by UC, or 250 μl of serum was mixed with precipitation solution, centrifuged and collected for subsequent RNA extraction.

EVs to be observed by transmission electron microscopy were suspended in 2.5% glutaraldehyde at 4 °C overnight. On the next day, vesicles were dropped in carbon-coated copper grids, stained with uranylacetate and imaged with a microscope (H7500 TEM, Japan). EVs were determined by Nanosight particle tracking analysis (Merkel Technologies Ltd., Israel, NTA 3.2 Dev Build 3.2.16). The concentrations of EV release were normalized as particle/cell to obtain net EV secretion rates.

### RNA extraction and qPCR

RNA was extracted using TRI reagent solution (Sigma) and RNeasy mini kit (Qiagen, Germany). RNA was also purified with miRCURY RNA Isolation Kits-Biofluids (Exiqon) from re-suspended EVs. Complementary DNA (cDNA) synthesis was performed using Transcriptor First Strand cDNA Synthesis Kit (Roche for mRNA and Novabio for miRNA). qPCR assay was performed using ABI 7900 T PCR System (Applied Biosystems, Foster City, USA). The sequences of the primers used for detection are provided in Additional file 1: Table S1.

### Immunoblotting

The cell and tissue lysates were extracted using RIPA lysis buffer containing protease and phosphatase inhibitor cocktail (NCM Biotech, Suzhou, China). Equivalent amounts of protein were electrophoresed on SDS-PAGE gels followed by transferring to PVDF membranes (Millipore, Billeria, MA). Antibodies against SNAP23 (Proteintech, China), TSG101, CD9 (Abcam, USA), Calnexin, Lin28a, Cyclin-D1, c-Myc, PKM2, p-PKM2 (Tyr105) (Cell Signaling Technology, USA) and ATP5A1, SDHA, CYTb, COX1 (ABclonal, Wuhan, China) were used for immunoblotted. β-Actin (Proteintech, China) served as the loading control. Protein bands were visualized and analyzed using the Odyssey Imaging System (LI-COR, USA) and Image J software.

### RNA interference and luciferase reporter assay

miRNA mimics, inhibitors and siRNAs were purchased from Shanghai GenePharma Company. According to the manufacturer’s instruction, an amount of 50 nM miRNA mimics, inhibitors or siRNAs were transfected into cells with Lipofectamine 3000 (Invitrogen, USA) to investigate the effect of let-7a on EV secretion.

Luciferase reporter assays were performed by co-transfecting HEK293 cells with let-7a mimic or ctrl with luciferase vectors (empty luciferase vector, luciferase vector containing wild-type target gene 3′-UTR or mutant-type target gene 3′-UTR) for SNAP23 using Lipofectamine 3000 and performing the Dual-Luciferase Reporter Assay System (Promega, USA) after 72 h.

### Establishment of stable cells

Lentiviral vectors containing shRNA SNAP23 (sh-SNAP23) and overexpressing SNAP23 were obtained from Shanghai Qihe Company, with shRNA non-targeted control (sh-NT) and empty vector (Vector) as controls. The designed target sequences were showed in Additional file 1: Table S1. The transduced CRC cells were selected with 5 μg/ml puromycin (Sigma, USA).

### Cell functional assays

Cell proliferation was assessed using a cell counting kit (CCK8; Dojindo, Japan). It was confirmed using a Gen5 microplate reader (BioTek, USA) by measuring the absorbance at 450 nm at 48 h. The EdU assay (RiboBio, Guangzhou, China) was also performed to detect the proliferation of CRC cells. Cell migration was determined by a wound healing assay. Cells were wounded by scratching lines with a pipette tip and then imaged after 48 h. Cell invasion was detected using transwell chambers (Corning, USA). Six hundred μl of DMEM containing 20% FCS was added in the lower chamber, and 100 μl of DMEM medium containing CRC cells was added to the upper chambers. The cells were incubated for 24 h and fixed with methyl alcohol and stained with 0.1% crystal violet.

### Bioenergetic assays

We used the Seahorse analyzer (Seahorse Bioscience, USA) to measure the mitochondrial activity of CRC cells. Briefly, cells were seeded into Seahorse miniplates (96 wells) overnight. After which cells were washed and exchanged in Seahorse assay buffer (adjust the pH to 7.4) in a 37 °C non-CO2 incubator for 1 h prior to assay, pharmaceutical compounds including oligomycin, FCCP, antimycin and rotenone or glucose, oligomycin and 2-DG were prepared to stressor mix at optimized concentration. The oxygen consumption rate (OCR) and extracellular acidification rate (ECAR) were then determined by Wave software. The values of OCR and ECAR were normalized to the number of cells per well. Cellular ATP levels were detected using the Cell-Glo ATP assay (Promega, USA).

### Fluorescence microscopy

Fluorescence microscopy was used to observe the subcellular localization and expression of SNAP-23 and mitochondrial in CRC cells. Cells were firstly added Mito-tracker™ Red CMXRos (Invitrogen™, USA) for 40 min. After it, cells were fixed with 4% paraformaldehyde for 15 min and then added with 0.2% Triton X-100 for 5 min and subsequently incubated with antibodies against SNAP-23 followed by incubation with Goat anti-Rabbit Secondary Antibody, Alexa Fluor 488 (Invitrogen™, USA). At last, the samples were imaged by confocal microscopy (Leica TCS SP8, Germany).

### Immunohistochemistry

Human CRC tissues were blocked and subsequently incubated with antibodies overnight at 4 °C, followed by incubation with the HRP-conjugated secondary detection antibodies (Dako Cytomation, USA) at room temperature for 30 min. The calculation of IHC staining scores was the same as the methods in our previous report [18]. Briefly, the staining intensity was independently scored by 2 pathologists blinded to the clinical data (0, no color; 1, weak; 2, moderate; 3, strong), and the percentage of positive cells (0, < 5%; 1, 6–25%; 2, 26–50%; 3, 51–75%; and 4, 76–100%) was semi-quantitatively assessed. The final scores (0–12) were then calculated by multiplying these 2 values. For analysis of clinical parameters, patients were divided into 4 subgroups based on this final score (negative, 0–2; weak, 3–4; moderate, 5–8; and strong, 9–12).

### Animal experiments

6-week-old female NSG mice were used for animal studies. All mice were injected into the back with 8.0 × 10^6^ SW480 cells expressing sh-NT or sh-SNAP23 to establish a xenograft model. Upon tumor xenografts reaching a volume of ~ 30 mm^3^, 3 μg of EVs derived from sh-NT SW480 cells were intravenously injected into mice through the tail vein twice a week. Each group had 4 mice and tumor volumes were measured twice weekly. After 4 weeks, all mice were sacrificed and tumor weights were calculated. All animal experiments were approved by the Shanghai General Hospital, Shanghai Jiao Tong University School of Medicine Animal Care and Use guidelines.

### Pathway analysis of data from the Cancer genome atlas

RNA-seq data of 456 colon cancer tissues and miRNA-seq data of 444 colon cancer tissues from TCGA Data Portal were obtained in March 2019. Four hundred forty-one samples with both RNA-seq data and miRNA-seq data were used to apply pathway analysis. The median value of let-7a was used to divide the samples into two groups. CAMERA performs a competitive test to identify whether the genes in the set are highly ranked in terms of differential expression relative to genes not in the set. The limma package and Hallmark gene sets curated by MSigDB was used to apply CAMERA to perform pathway analysis. Gene sets with adjusted *p*-value below 0.05 were considered significantly enriched.

### Statistical analysis

Each result was repeated from three independent experiments and presented as mean ± SEM. Two-tailed unpaired and paired Student’s t-test and x^2^-test were used to determine statistical comparisons, and *p*-value < 0.05 was considered statistically significant.

## Results

### Let-7a was markedly upregulated in the serum EVs of CRC patients

**Fig. 1:**
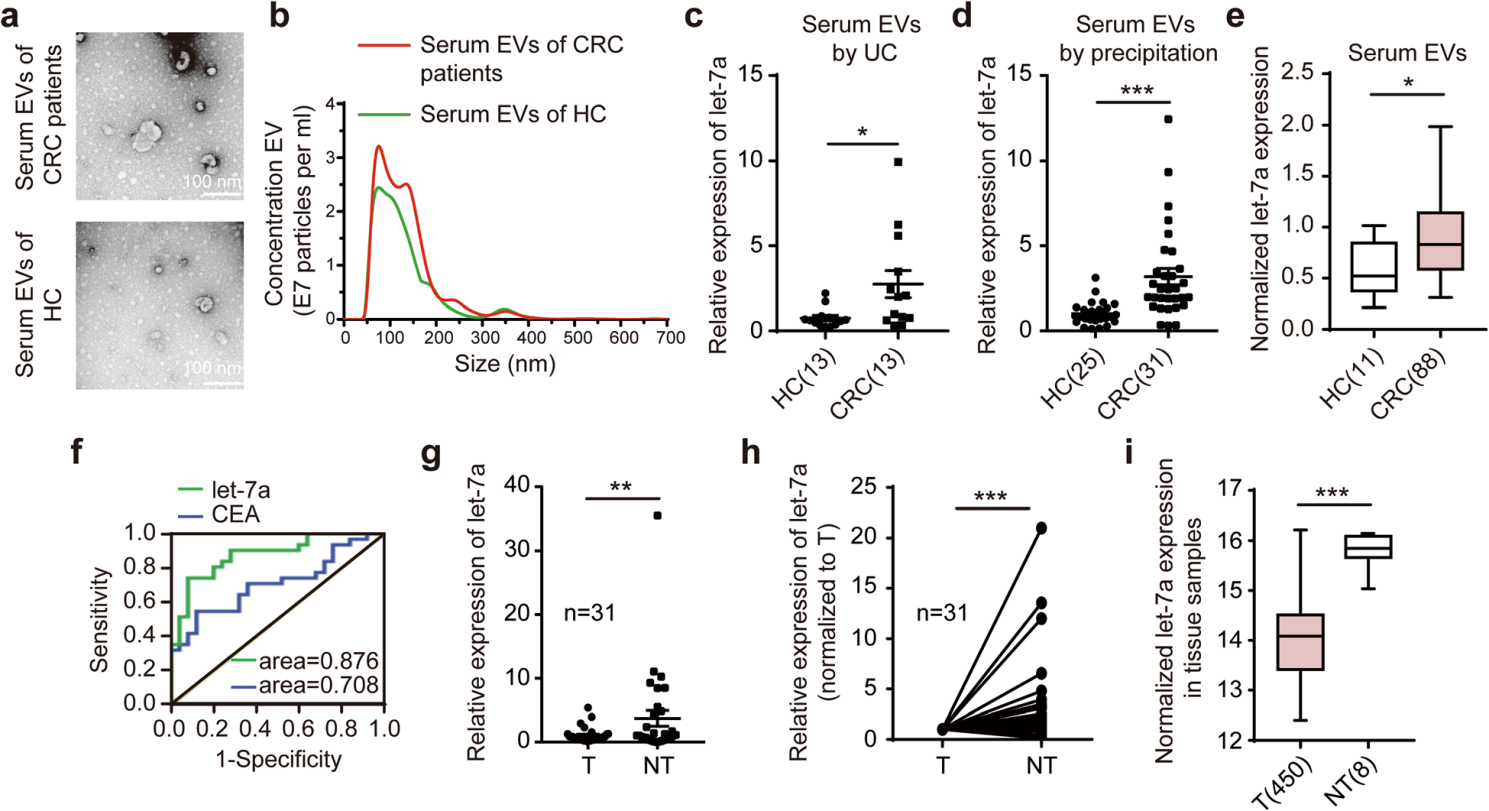
Let-7a is enriched in serum-derived EVs from CRC patients. EVs isolated from serum of CRC patients or heathy controls (HC) using ultracentrifugation (UC) were confirmed by transmission electron microscopy (**a**) and Nanosight particle tracking analysis (**b**). Scale bar, 100 nm. **c** qPCR analysis of let-7a from serum EVs of CRC patients (*n* = 13) and HC (n = 13) isolated by UC methods, with U6 as the loading control. **d** qPCR analysis of let-7a from serum EVs of CRC patients (*n* = 31) and HC (*n* = 25) isolated by precipitation methods, with U6 as the loading control. **e** Comparative analysis of serum EV let-7a in CRC patients (*n* = 88) and HC (*n* = 11) from the Database: GSE40247. **f** ROC curves for the diagnostic values of serum EV let-7a and CEA in CRC patients (*n* = 31) and HC (*n* = 25). **g**, **h** Let-7a expression in CRC patient tumor (T) tissue with paired adjacent non-tumor (NT) samples (*n* = 31). (i) Let-7a expression between CRC (T, *n* = 450) and non-tumor (NT, *n* = 8) tissue from TCGA database are shown. Data represent the mean ± SEM of at least three independent experiments. *p < 0.05, **p < 0.01 and ***p < 0.001

Our preliminary miRNA-sequence data from serum-derived EVs of CRC patients indicates the significant differential expression of let-7a. Considering the importance of EV-derived let-7a in the progression of colorectal, we first explored the expression levels of let-7a in serum EVs. We isolated the serum EVs from CRC patients and HC by UC methods, and then confirmed their identity by electron microscopy and Nanosight analysis (Fig. 1a-b). We found the expression of serum EV let-7a from CRC patients (*n* = 13) was upregulated compared to that from HC (n = 13) (Fig. 1c). We also isolated EVs from other serum samples using precipitation for an easier and cheaper enrichment for miRNA analyses [16, 19]. We confirmed elevated expression levels of serum EV let-7a in CRC patients (*n* = 31) compared with HC (*n* = 25) (Fig. 1d), consistent with the miRNA microarray from the Database: GSE40247 (Fig. 1e). Furthermore, we found that the diagnostic efficacy of serum EV let-7a was better to that of the traditional tumor marker CEA (Fig. 1f). However, serum EV let-7a showed no significant changes amongst the parameters of age, sex, distant metastasis, or tumor stages (Additional file 1: Table S2). Interestingly, we discovered that let-7a expression was reduced in tumor tissue (Fig. 1g, h), which was confirmed by TCGA database (Fig. 1i).

### Let-7a regulates EV secretion in CRC cells

**Fig. 2:**
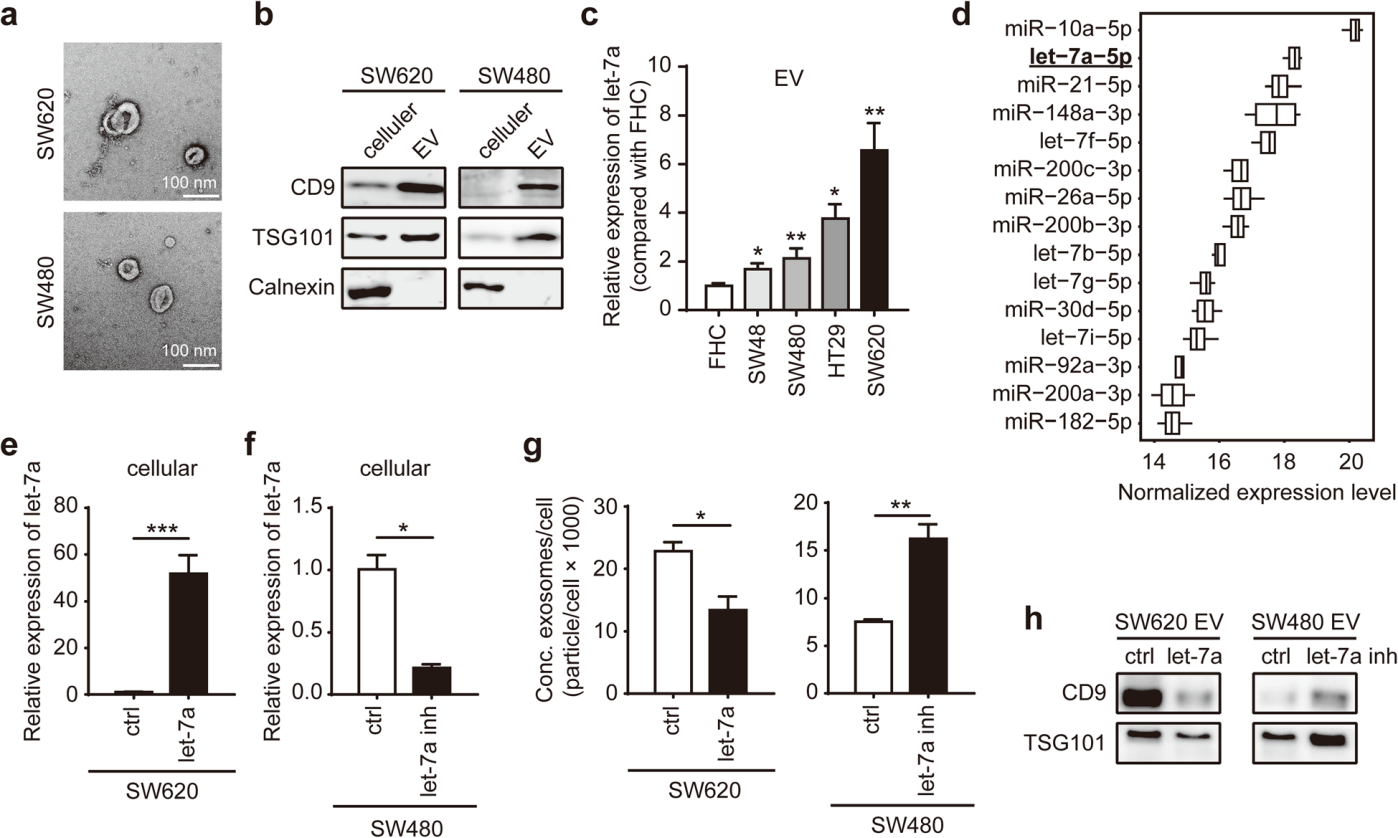
Let-7a regulates EV secretion in CRC cells. **a** EVs released by different CRC cells were detected by electron microscopy. Scale bar, 100 nm. **b** Western blot analysis of indicated proteins in total cell lysate or EVs from CRC cells, with Calnexin as the negative controls for EVs. **c** qPCR analysis of let-7a expression in EVs derived from FHC cell and CRC cell lines. **d** Top 15 most abundant miRNAs identified in CRC cell-derived EVs from the Database: GSE125905. **e** qPCR analysis of let-7a expression in SW620 transfected with ctrl or let-7a mimic. **f** qPCR analysis of let-7a expression in SW480 transfected with ctrl or let-7a inhibitor. **g** Effect of let-7a mimic or inhibitor on EV secretion from CRC cells using Nanosight particle tracking analysis. **h** Effect of let-7a mimic or inhibitor on EV proteins from CRC cell-derived EVs. Data represent the mean ± SEM of at least three independent experiments. *p < 0.05, **p < 0.01 and ***p < 0.001

EVs released by different CRC cells were identified by electron microscopy (Fig. 2a). The indicated proteins in total cell lysate or EV from SW480 and SW620 were analyzed by Western blot (Fig. 2b). We evaluated the intracellular let-7a expression and extracellular let-7a expression in a normal colon epithelial cell line (FHC) and four CRC cell lines (SW48, SW480, HT29 and SW620) (Fig. 2c, Additional file 2: Fig. S1a). It indicates that let-7a expression levels were significantly decreased in CRC cells compared with FHC cell (Additional file 2: Fig. S1a). However, let-7a levels from EVs were markedly increased in CRC cells in comparison with FHC cell (Fig. 2c). It strongly suggests that the intracellular let-7a expression is most likely affected by EV secretion. Importantly, the lower endogenous levels and higher exogenous expression of let-7a were found in SW620 cells, a metastatic derivative from the same patient from which SW480 cells were derived. Consistent with our experimental results, we found let-7a was enrichment in CRC cell-derived EVs from the GSE125905 dataset (Fig. 2d).

Thus, we hypothesized that EV secretion might regulate intracellular let-7a expression of CRC cells by releasing it into the extracellular fluids. SW620 and SW480 cells were transfected with miRNA mimic or inhibitor of let-7a. The expression of let-7a after transfection with miRNA mimic or inhibitor was confirmed by qPCR (Fig. 2e, f). It was confirmed via Nanosight analysis that the particle number of EVs secreted by SW620 cells transfected with let-7a mimic was decreased. On the other hand, inhibition of let-7a led to an enhanced ability of EV secretion in SW480 cells (Fig. 2g). We also extracted proteins of EVs isolated from an equal number of cells. The effects of let-7a mimic or inhibitor on EV proteins from these two cell lines also verified similar results (Fig. 2h).

### Let-7a-promoted EV release in CRC cells is dependent on SNAP23

**Fig. 3:**
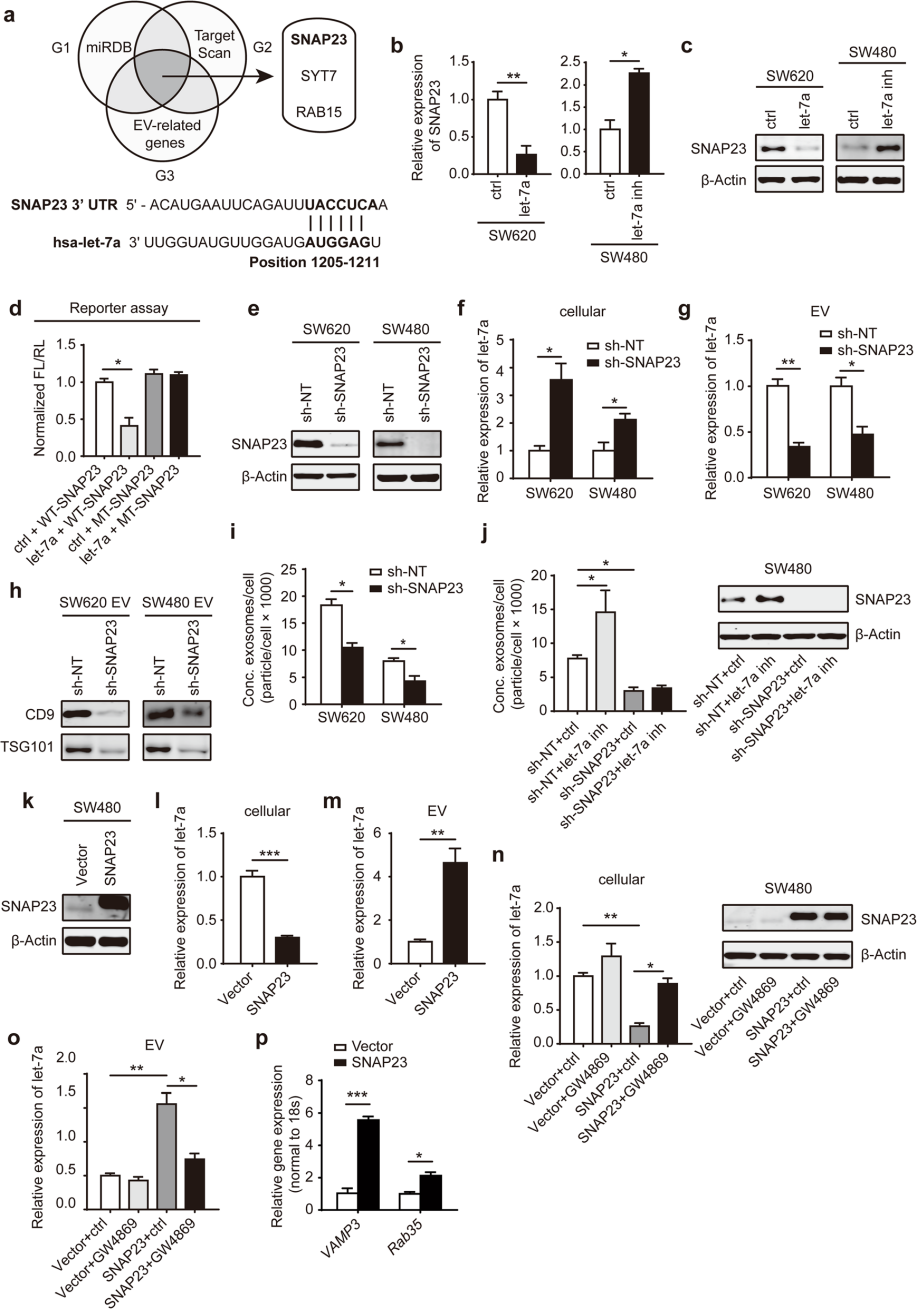
SNAP23 directly regulates the secretion of let-7a in EVs. **a** Venn diagram for selecting candidate target genes of let-7a using the three indicated data sets. G1, G2 predicted targets from miRDB (*n* = 990 genes) and Targetscan (*n* = 1207 genes); G3, EV secretion-related genes (*n* = 31). Summary of let-7a target sites in the 3’ UTRs of SNAP23 in the below. qPCR (**b**) and western blot analysis (**c**) of SNAP23 were determined in CRC cells expressing let-7a mimic or inhibitor, with U6 or β-Actin as the loading control. **d** Luciferase reporter assays confirmed the direct binding of let-7a to SNAP23. WT, wild type; MT, mutant. **e** Western blot analysis of SNAP23 in sh-NT and sh-SNAP23 CRC cells. **f** qPCR of intracellular let-7a from sh-NT and sh-SNAP23 CRC cells. **g** qPCR of extracellular let-7a in EVs from sh-NT and sh-SNAP23 CRC cells. **h** Western blot analysis of indicated proteins in EVs from CRC cells transfected with sh–NT and sh-SNAP23. **i** The ability of sh-SNAP23 CRC cells on EV secretion dramatically decreased compared to sh-NT CRC cells. **j** The effect on EV secretion (left) and SNAP23 expression (right) of SW480 sh-NT and sh-SNAP23 cells treated with ctrl or let-7a inhibitor. **k** Western blot analysis of SNAP23 in SW480 cells stably overexpressing empty vector and SNAP23. qPCR of intracellular let-7a (**l**) and extracellular let-7a (**m**) in SW480 cells overexpressing vector and SNAP23. **n** qPCR of intracellular let-7a (left) and western blotting of SNAP23 (right) from SW480 overexpressing vector and SNAP23 treated with vehicle ctrl or 10 μM GW4869. **o** qPCR of extracellular let-7a in EVs from SW480 overexpressing vector and SNAP23 treated with vehicle ctrl or GW4869. **p** qPCR analysis of related genes in SNARE complex from SW480 cells overexpressing vector and SNAP23. Data represent the mean ± SEM of at least three independent experiments. *p < 0.05, **p < 0.01 and ***p < 0.001

We next sought to elucidate the targets of let-7a in EV secretion, which initially involved integrating three individual gene sets, as follows: two bioinformatics sets (G1, miRDB; G2, Targetscan) and an EV secretion-related gene set (G3) [20]. Upon undertaking a combinatorial analysis, 3 genes (*SNAP23, SYT7 and RAB15*) were identified (Fig. 3a). As the oncogenic role of SNAP23 in EV secretion has been reported [21], we select SNAP23 to further investigation and found that the expression of SNAP23 negatively correlated with the let-7a levels in CRC cell lines (Additional file 2: Fig. S1a, b). Firstly, we confirmed that mRNA and protein expression levels of SNAP23 could be downregulated in SW620 cells transfected with let-7a mimic, or upregulated in SW480 cells transfected with inhibitor (Fig. 3b, c). Then, to address whether let-7a directly regulated SNAP23, a luciferase reporter assay was performed. The luciferase activity of wild-type reporter plasmids was significantly suppressed by let-7a mimic, whereas the luciferase activity of mutant-type reporter plasmids remained unchanged (Fig. 3d). In addition, we established the SW480 and SW620 cells stably expressing shRNAs targeting SNAP23 to knockdown SNAP23 expression (Fig. 3e, Additional file 3: Fig. S2a). There was a significant upregulation of let-7a levels in sh-SNAP23 CRC cells, with no changes in the levels of two unrelated miRNAs (Fig. 3f, Additional file 3: Fig. S2b). However, extracellular let-7a levels from sh-SNAP23 CRC cells showed a markedly downregulation (Fig. 3g, Additional file 3: Fig. S2c). Indicated EV protein expression and particle number of EVs were significantly decreased in CRC cells expressing sh-SNAP23 (Fig. 3h, i). After inhibiting the let-7a expression of SW480 cells transfected with sh-SNAP23, EV secretion was not activated (Fig. 3j).

We also established CRC cells stably overexpressing SNAP23 to further investigation (Fig. 3k). Forced expression of SNAP23 in SW480 CRC cells led to decreased intra- (Fig. 3l) and increased extracellular expression of let-7a (Fig. 3m). When SW480 cells overexpressing empty vector and SNAP23 were treated with a chemical inhibitor of neutral sphingomyelinases, GW4869, which has been found to be required for the release of vesicles from MVBs [22, 23], we observed that the decreased intracellular levels of let-7a in SW480 cells overexpressing SNAP23 were rescued by the addition of GW4869 (Fig. 3n). The increased extracellular levels of let-7a were also rescued in SW480 cells overexpressing SNAP23 with the treatment of GW4869 (Fig. 3o). SNAP23 is a t-SNARE molecule that contributes to release EV including v-SNARE VAMP and Ras-related protein Rab-35 (RAB35). We also found that t-SANRE SNAP23 could upregulate the expression of VAMP3 and RAB35, which promote EV secretion (Fig. 3p). Overall, these results provide experimental evidence that SNAP23 is a direct downstream target of let-7a and mediates EV secretion to keep steady-state levels of let-7a.

### SNAP23 is critical for let-7a-mediated cell growth

**Fig. 4:**
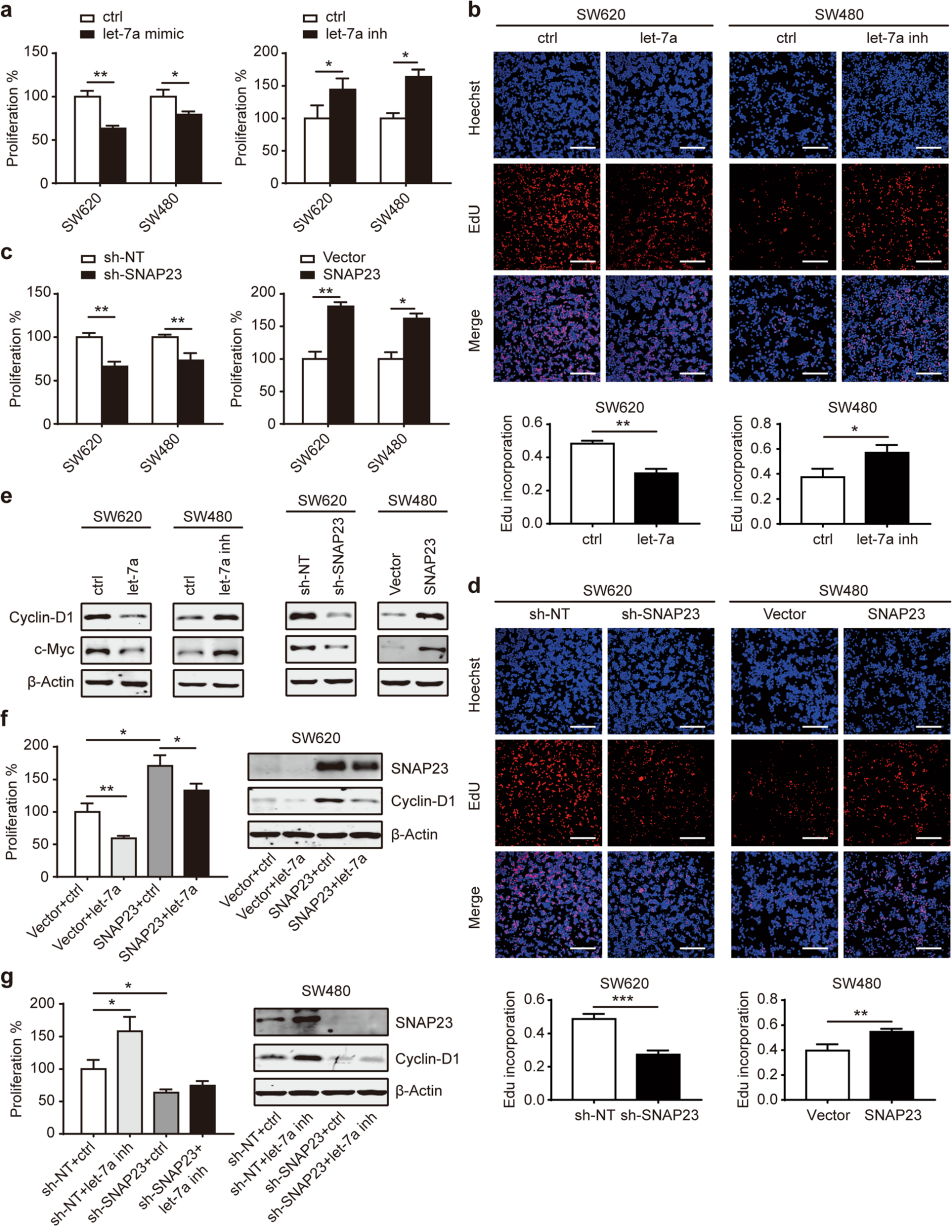
Let-7a/SNAP23 suppresses the proliferation of CRC cells. The proliferation of SW620 and SW480 CRC cells transfected with let-7a mimic or let-7a inhibitor was detected by CCK-8 (**a**) and EdU assays (**b**). Scale bar, 200 μM. The proliferation of CRC cells transfected with sh-SNAP23 or overexpressing SNAP23 was detected CCK-8 (**c**) and EdU assays (**d**). Scale bar, 200 μM. **e** Western blot analysis of cell cycle related proteins in SW620 and SW480 after the transfection of let-7a mimic or inhibitor and sh-SNAP23 or overexpressing SNAP23. **f** Cell proliferation by CCK-8 (left) and western blot of SNAP23 and Cyclin-D1 (right) in SW620 cells overexpressing vector and SNAP23 treated with ctrl or let-7a mimic. **g** Cell proliferation by CCK-8 (left) and western blot of indicated proteins (right) was shown in SW480 sh-NT and sh-SNAP23 treated with ctrl or let-7a inhibitor. Data represent the mean ± SEM of at least three independent experiments. *p < 0.05, **p < 0.01 and ***p < 0.001

Since much evidence has demonstrated that let-7a functions as a tumor suppressor in various tumors including CRC [14, 24], we further determined whether SNAP23 is critical for let-7a-regulated tumor growth. Consistent with the previous reports, the proliferation of CRC cells transfected with let-7a mimic was dramatically decreased (Fig. 4a, b). And the suppressed let-7a showed increased proliferation (Fig. 4a, b). On the contrary, the inhibition of SNAP23 decreased cell proliferation and overexpressed SNAP23 increased cell proliferation in CRC cells (Fig. 4c, d). Let-7 has been reported to directly binding to the 3’UTRs of downstream gene cyclin-D1, which promotes cell cycle progression and oncogenesis [25, 26]. We next determined that let-7a could downregulated cyclin-D1 and c-Myc expression (Fig. 4e). On the contrary, SNAP23 upregulated the expression of cell cycle related proteins in CRC cells (Fig. 4e). It suggests that let-7a/SNAP23 is involved in Wnt/β-catenin signaling pathway, such as those encoding cyclin-D1 and c-Myc genes.

**Fig. 5:**
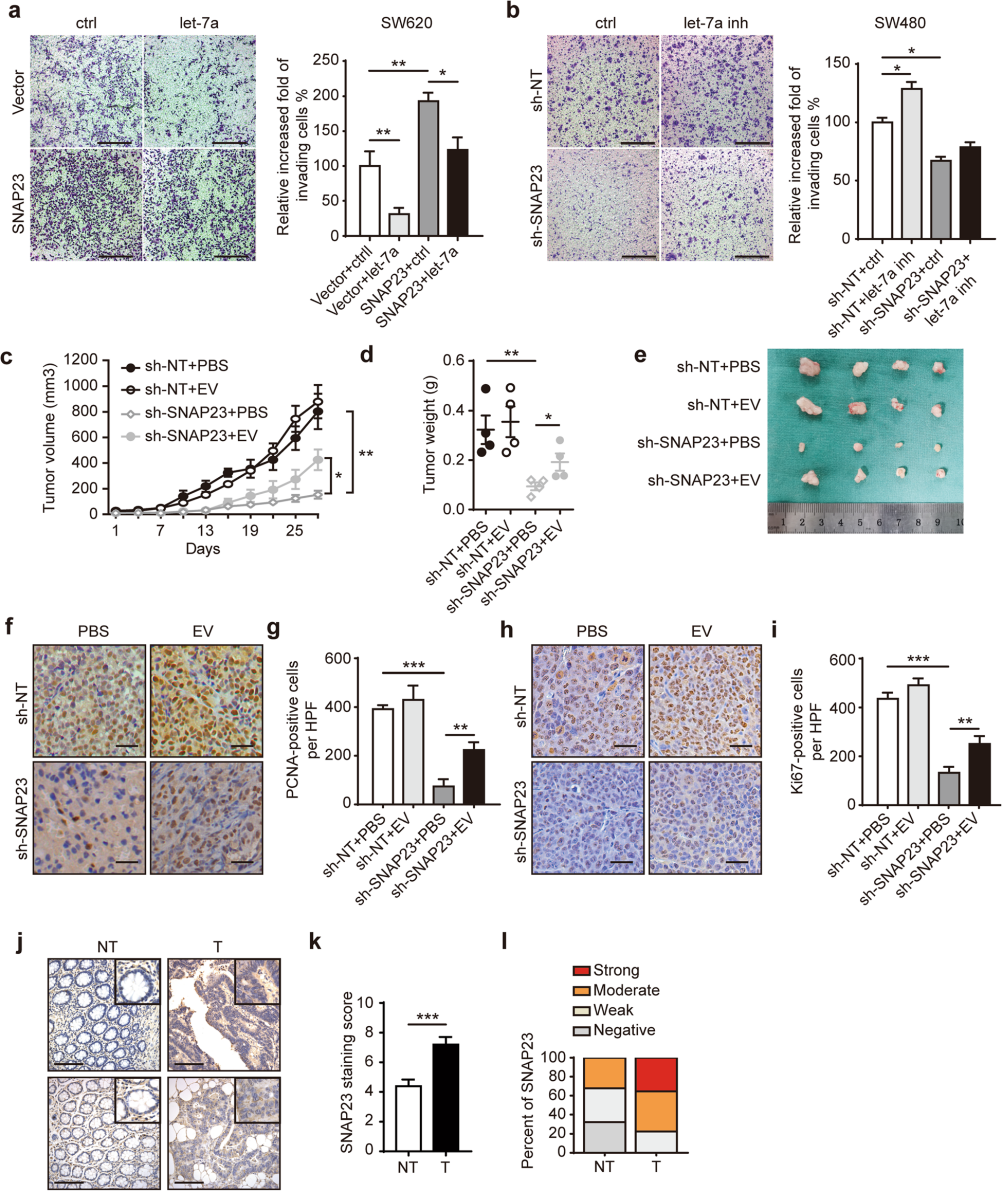
Downregulation of EV secretion via let-7a/SNAP23 axis inhibits tumor growth. **a** Cell invasion by transwell assay in SW620 cells overexpressing vector and SNAP23 treated with ctrl or let-7a mimic. Scale bar, 200 μM. Original magnification: 200×. **b** Cell invasion in SW480 sh-NT and sh-SNAP23 treated with ctrl or let-7a inhibitor. Scale bar, 200 μM. Original magnification: 200×. **c** Upon SW480 sh-NT and sh-SNAP23 tumor xenografts reaching a volume of ~ 30 mm^3^, 3 μg of EVs derived from sh-NT SW480 cells or PBS were intravenously injected into NSG mice through the tail vein twice a week. Tumor sizes were measured twice weekly. **d** Tumor weights were determined at the experimental end-point (4 weeks). **e** Representative images of xenograft tumors suggested that knockdown of SNAP23 significantly reduced tumor sizes and weights. However, an injection of EVs from CRC cells partially rescued tumor sizes and weights in sh-SNAP23 groups. **f**, **h** Representative images of xenograft tumors that were subjected to PCNA and Ki67 staining. Scale bar, 40 μM. **g**, **i** The numbers of positive cells/high power field (HPF) were shown in groups. **j** Representative IHC for SNAP23 in CRC tumor tissue (T) compared with adjacent normal tissue (NT). Scale bar, 100 μM. IHC staining scores (**k**) and percentage of SNAP23 levels (**l**) from CRC patients (*n* = 31) showed that SNAP23 expression in CRC groups (T) was higher than in normal groups (NT). Data represent the mean ± SEM of at least two independent experiments. *p < 0.05, **p < 0.01 and ***p < 0.001

In complementary loss-of-function assays, the obviously suppressed proliferation in SW620 treated with let-7a mimic was partly rescued in SW620 cells overexpressing SNAP23 (Fig. 4f). In addition, enhanced proliferation in SW480 treated with let-7a inhibitor was obviously restrained when SNAP23 was knocked down (Fig. 4g). More importantly, the effect of let-7a inhibitor on the increase of Cyclin-D1 was offset by SNAP23 knockdown (Fig. 4g). The migration and invasion of SW620 and SW480 cells with different treatments showed similar results (Fig. 5a, b, Additional file 4: Fig. S3a-d). Interestingly, the combination with the let-7a mimic and sh-SNAP23 in SW620 cells greatly suppressed CRC cell proliferation, migration and invasion (Additional file 4: Fig. S3e-g). Collectively, these data demonstrate that SNAP23 is essential for let-7a-mediated tumor cell growth.

Given that let-7a-SNAP23 axis significantly inhibited the tumor growth in vitro, we next using SW480 cells with stable SNAP23 depletion to determine the antitumor activity in vivo. The knockdown of SNAP23 could inhibit the tumorigenicity of CRC cells in NSG mice (Fig. 5c-e). In addition, the tumor sizes and weights of sh-SNAP23 SW480 bearing mice were partially rescued with the injection of sh-NT SW480-derived EVs (Fig. 5c-e). Consistent with these data, suppressed SNAP23 expression showed reduced numbers of proliferative PCNA^+^ and Ki67^+^ cells and increased EV injection partially elevated the numbers of positive cells (Fig. 5f-i). These data suggest that SNAP23 regulates EV secretion to promote tumorigenesis. Furthermore, the clinical expression of SNAP23 was investigated by Immunohistochemistry (IHC) analysis on paired CRC tumors and adjacent normal tissue samples (*n* = 31) (Fig. 5j). It revealed that SANP23 expression was significantly upregulated in tumor samples using staining scores (Fig. 5k). Approximately 77.4% of CRC exhibited strong (35.5%, *n* = 11) or moderate (41.9%, *n* = 13) SNAP23 staining, while weak (35.5%, *n* = 11) or negative (32.2%, *n* = 10) staining was observed in matched adjacent normal tissue (Fig. 5l). However, SNAP23 expression showed no significant changes amongst the parameters of clinical pathologic characteristics (Additional file 1: Table S3).

### Let-7a regulates the mitochondrial metabolic phenotype via SNAP23

**Fig. 6:**
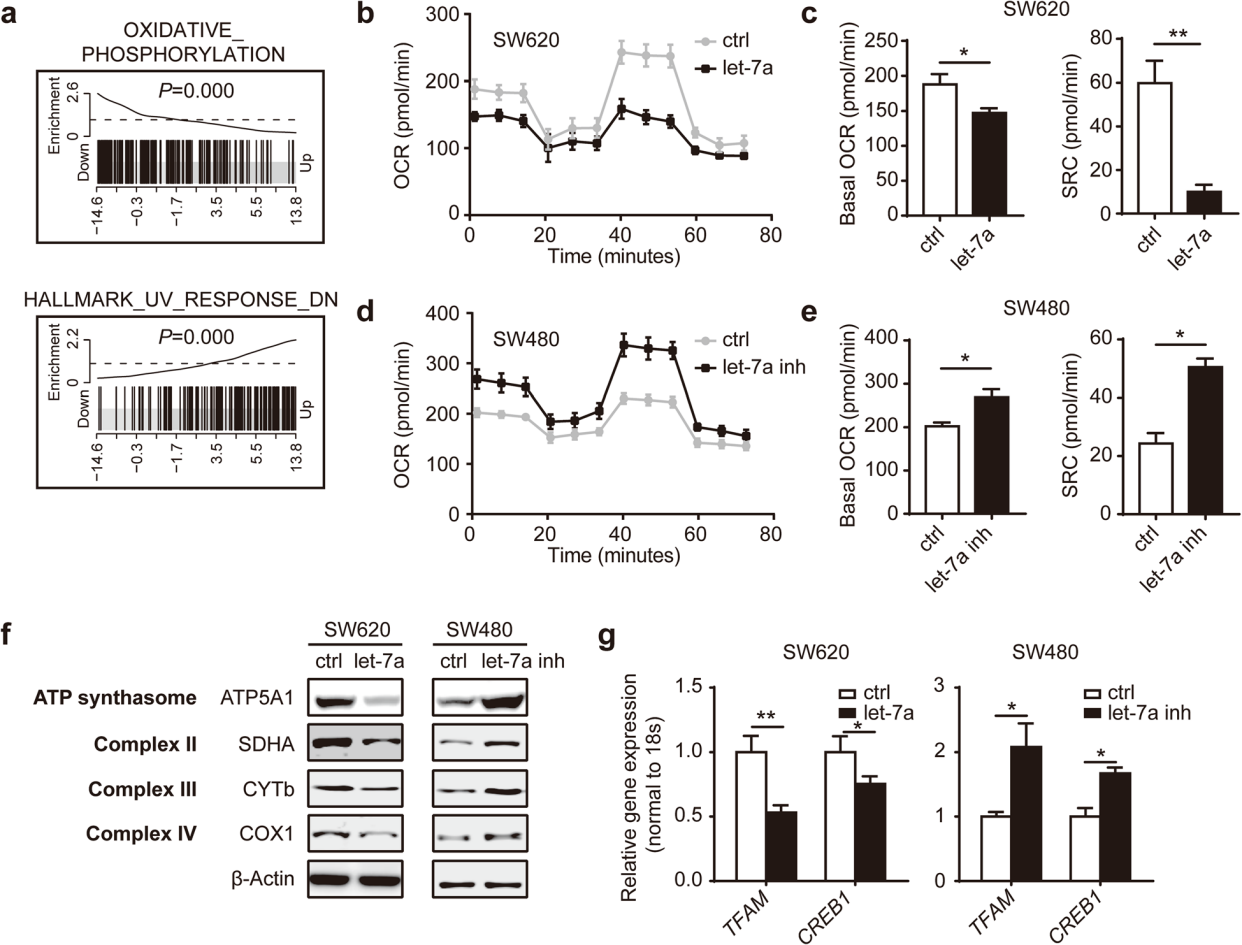
Let-7a suppresses mitochondrial OXPHOS in CRC cells. **a** Barcode enrichment plots of the top two enriched gene sets identified by CAMERA between “let-7a low” and “let-7a high” subgroups in the TCGA CRC cohort based on hallmark gene sets are shown as an example of maximum possible enrichment. Oxygen consumption rate (OCR) was assayed using the Seahorse analyzer (Seahorse Bioscience) in SW620 transfected with let-7a mimic (**b**) or SW480 transfected with let-7a inhibitor (**d**) with the treatment of oligomycin, FCCP and a mix of antimycin A and rotenone. **c**, **e** Basal OCR and spare respiratory capacity (SRC) were measured in SW620 and SW480 cells. **f** Western blot analysis of mitochondrial OXPHOS complexes in SW620 and SW480 after the transfection of let-7a mimic or inhibitor. **g** qPCR analysis of genes mediating OXPHOS in SW620 and SW480 after the transfection of let-7a mimic or inhibitor. Data represent the mean ± SEM of at least three independent experiments. *p < 0.05 and **p < 0.01

Let-7 family miRNAs have been reported to suppress glycolysis through the targeted genes *Lin28*, which could regulate the translation of mRNAs for several metabolic enzymes [27]. However, the mechanisms underlying alteration of OXPHOS, with most cancers retaining mitochondrial function to facilitate the dynamic interplay between OXPHOS and glycolysis, remain unclear in cancers, even more obscure in CRC. Interestingly, CAMERA using hallmark gene sets generated by MSigDB showed that the “let-7a low” patient cohort was enriched for OXPHOS (Fig. 6a). Furthermore, two CRC cell lines after transfection with let-7a mimic or inhibitor were selected to determine the metabolic phenotype by the Seahorse analyzer. The basal oxygen consumption rate (OCR) and spare respiratory capacity (SRC) were decreased by let-7a mimic (Fig. 6b, c) or increased by let-7a inhibitor (Fig. 6d, e). We also tested that let-7a could suppress glycolysis in CRC cells (Additional file 5: Fig. S4a, b). To further test this, we determined OXPHOS protein expression levels that a reduction in ATP5A1, knows as ATP synthasome, complex II SDHA, complex III CYTb and complex IV COX1 from SW620 cells transfected with let-7a mimic (Fig. 6f). On the other hand, inhibition let-7a expression of SW480 cells showed the improved indicated protein expression (Fig. 6f). Consistent with the previous results, genes encoding factors that promote OXPHOS, namely mitochondrial transcription factor A (*TFAM*) and cyclic AMP-responsive element-binding protein 1 (*CREB1*) were also decreased in SW620 cells expressing let-7a mimic or increased in SW480 cells expressing let-7a inhibitor (Fig. 6g).

**Fig. 7:**
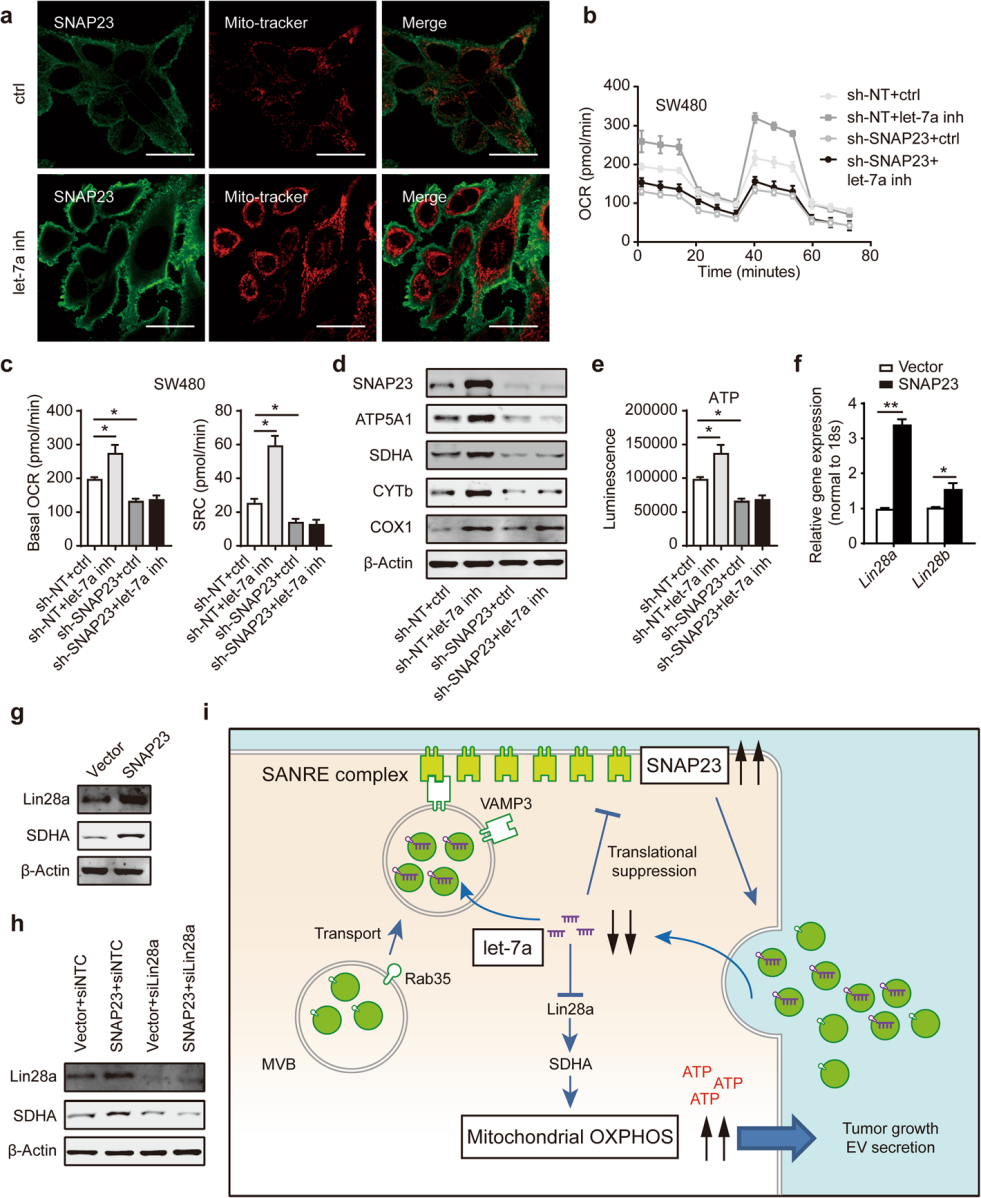
Let-7a-regulated cancer cell OXPHOS is dependent on SNAP23. **a** Staining of SW480 cells treated with ctrl or let-7a inhibitor with Mito-tracker (red) and anti-SNAP23 (green). Scale bar, 10 μm. **b**, **c** SW480 sh-NT and sh-SNAP23 cells were transiently transfected with ctrl or let-7a inhibitor, then subjected to measure basal OCR and SRC using Seahorse analyzer. **d** Western blot analysis of indicated protein expression of SW480 sh-NT and sh-SNAP23 cells treated with ctrl or let-7a inhibitor. **e** Cellular ATP levels were measured in SW480 sh-NT and sh-SNAP23 cells treated with ctrl or let-7a inhibitor. **f** qPCR analysis of indicated genes in SW480 cells overexpressing Vector and SNAP23. **g** Western blot analysis of Lin28a and SDHA protein expression in SW480 cells overexpressing Vector and SNAP23. **h** Western blot analysis of Lin28a and SDHA in SW480 cells overexpressing Vector and SNAP23 transiently transfected with siNTC or siLin28a. **i** Model for the role of let-7a in the EV secretion and mitochondrial OXPHOS via SNAP23 in CRC. Data represent the mean ± SEM of at least three independent experiments. *p < 0.05, and **p < 0.01

Recently, it has been reported that the enhanced aerobic glycolysis could active EV secretion via PKM2-SNAP23 axis [21]. However, few studies have explored the link between the increased mitochondrial energy and enhanced EV secretion in tumor cells. We analyzed the expression and localization of SNAP23 in CRC cells. Confocal microscope showed an increased expression of SNAP23 (green) and enhanced mitochondrial biogenesis (red) in SW480 cells transfected with let-7a inhibitor (Fig. 7a). Hence, we determined the metabolic phenotype in SW480 cells expressing sh-SNAP23 transfected with let-7a inhibitor or ctrl inhibitor. Seahorse analysis revealed that knockdown SNAP23 diminished the promoting effect of let-7a on cell OXPHOS (Fig. 7b, c). Mitochondrial OXPHOS complexes proteins were not activated after inhibiting let-7a expression in SW480 cells stably transfected with sh-SNAP23 (Fig. 7d). Given that the protein expression of ATP5A1, as a functional ATP production, showed the most significant change, we measured cellular ATP levels, and as a result that SW480 cells treated with let-7a inhibitor enhanced the ATP production (Fig. 7e). However, downregulated SNAP23 expression eliminate the effect on an enhanced ability of ATP synthesis by the inhibition of let-7a (Fig. 7e). It has been found that let-7 increased OXPHOS via Lin28/SDHA axis [27, 28]. Since SNAP23 could also downregulate intracellular let-7a expression (Fig. 3f, l), we further investigated the expression of Lin28 family in CRC cell overexpressing SNAP23. Interestingly, the enhanced expression of Lin28a, Lin28b and SDHA were determined in SNAP23 overexpressed SW480 cells (Fig. 7f, g). Because it has been reported that Lin28a/Sdha axis could promote OXPHOS [28], we next used siRNA to suppress the expression of Lin28a. The increased SDHA expression was not activated in SNAP23 overexpressed SW480 cells treated with the inhibition of Lin28a (Fig. 7h). It supports our hypothesis that SNAP23 in turn downregulates let-7a expression to increase OXPHPS through Lin28/SDHA axis. Taken together, our observation showed a novel mechanism by which let-7a/SNAP23 regulates OXPHOS that are involved in EV secretion (Fig. 7i).

## Discussion

It has been well established that miRNA let-7 family, first identified in *Caenorhabditis elegans*, is essential pathological events in tumorigenesis and progression of various human cancers [29]. As tumor suppressors, let-7 miRNAs inhibit oncogenes expression including c-Myc, K-ras, HMGA2 and cell cycle factors [30]. Loss expression of let-7 relieves suppression of these oncogenes facilitating tumor growth and metastasis. However, the functional role of let-7 miRNA in EVs remains unclear.

In our study, we first determined that let-7a was markedly upregulated in the serum EVs of CRC patients compared to health groups. And we also determined that serum EV let-7a could be an effective blood marker for tumor detection, suggesting the clinical importance of our findings. However, we confirmed the reduced let-7a expression in CRC tissue, consistent with early results that let-7a is known to be downregulated in various human malignancies including CRC [29, 31]. These results prompted us to seek the causes of the difference between let-7a expression levels in tissue and serum, which have never been reported before. A previous study found that the regulation of the intracellular levels of miR-200 family involved their secretion in EVs by protein kinase C to maintain miR-200 steady-state levels [23]. Therefore, we assume a specific link of let-7a to EV secretion in tumors. To support this, we show the significant difference between intra- and extra-cellular expressions of let-7a in CRC cell lines. SW620 with high malignant degree decreased endogenous and increased exogenous expression of let-7a compared to SW480. Furthermore, our data indicated that let-7a regulated EV secretion in CRC cells (Fig. 2g, h). As mentioned above, let-7a not only inhibits tumorigenesis but also prevents the secretion of EVs.

The biogenesis and release of EVs are regulated by a variety of molecules, which involves the transport of MVBs and their docking and fusion with the plasma membrane. It has previously been reported that Rab small GTPases, including Rab27a and Rab27b, participate in regulating different steps of EV secretion [22, 32]. SNAP23 is a t-SNARE molecule mediating the fusion process that contributes to release intraluminal vesicles [20, 33]. To investigate the target genes of let-7a that inhibit EV secretion, three individual gene sets were combined analyzed, and three candidate genes, *SNAP23, SYT7 and RAN15* were selected. Given that SNAP23 is widely expressed in cell types and plays a big role in various kinds of carcinomas [20, 21], we choose SNAP23 to further investigation. Let-7a directly regulated SNAP23 was determined using a luciferase reporter assay. Next, we inhibited let-7a expression in SW480 cells expressing sh-SNAP23, that the enhanced ability of EV secretion was lost. Several studies have demonstrated that the suppression of cancer-derived EVs may have therapeutic value by inhibiting cancer proliferation and metastasis [34, 35].

We further investigated the effects of SNAP23 to cell growth that downregulated SNAP23 expression suppressed the proliferation, migration and invasion of CRC cells. Importantly, sh-SNAP23 CRC cells with overexpressed let-7a had a more significant impact on the inhibition of cell growth. Furthermore, animal experiments showed that the tumor xenografts of NSG mice injected with EVs increased the tumor size and weight of the SNAP23-depleted SW480 xenografts. Besides, elevated SNAP23 expression in CRC tissue has been confirmed compared to adjacent normal samples. Overall, our results demonstrated that let-7a/SNAP23 axis could provide not only effective tumor biomarkers but also novel targets for tumor therapeutic strategies.

Another key finding of this work was that let-7a suppressed mitochondrial OXPHOS in CRC cells dependently on SNAP23, as well as the regulation of EV secretion. The “Warburg effect”, a defining feature of cancer cells to employ a modified metabolic program with increased glycolytic activity and lactate secretion, even when oxygen is present, facilitates cell energy needs to support tumor growth [36]. However, this enhanced aerobic glycolysis of cancers doesn’t occur from a consequence of defective mitochondrial respiration, with most cancers retaining mitochondrial function to facilitate the dynamic interplay between OXPHOS and glycolysis [37]. Our previous study has reported that Toll-like receptor 2 could augment both OXPHOS and glycolysis to promote tumor growth [18]. It has also been established that Lin28 and let-7 family enhanced the translation of mRNAs for several metabolic enzymes, thereby increasing not only OXPHOS but also glycolysis [27]. In another study, let-7 was reported to facilitate glycolysis while inhibiting OXPHOS process in hepatoma cells [14]. Therefore, a provocative question remains to be answered to understand more adequately the role of let-7-regulated mitochondrial metabolic reprogramming. Apart from it, the increased secretion of EVs is another phenomenon observed during tumorigenesis. But few studies have explored the link between the metabolic reprogramming and active EV secretion in tumor cells. We confirmed that let-7a significantly suppressed mitochondrial OXPHOS and ATP synthesis in CRC cells. Furthermore, the mechanism of let-7a-regulated OXPHOS was been found to be closely related to SNAP23. Although several studies have reported that let-7a could directly downregulated PKM2 [38, 39], we found no obvious change of PKM2 with overexpression of let-7a in CRC cells (Additional file 5: Fig. S4c). Because SNAP23 could in turn downregulate the let-7a expression, we assumed that SNAP23 promoted OXPHOS by regulating the lin28/let-7a pathway. Previous studies suggest that Lin28a/Sdha axis could promote OXPHOS in macrophages [28]. We found SNAP23 upregulated Lin28/SDHA axis in CRC cells. Interestingly, SNAP23 has been observed to locate in the mitochondrial membrane and facilitate the fatty acids into mitochondria for β-oxidation [40–42]. SNAP23 might co-localize with plasma membrane and mitochondria, which clarifies the reason that the inhibition of it eliminates the effect of enhanced OXPHOS.

Since cancer cells released large amounts of intracellular let-7a miRNAs to extracellular fluids, the function of extracellular let-7a absorbed into recipient cells is still worth investigating. Several studies reported that extracellular let-7a suppressed the growth of tumor cells [43, 44]. However, the tumor growth of sh-SNAP23 SW480 bearing mice was partially rescued with the injection of sh-NT SW480-derived EVs (Fig. 5). Let-7a was enriched in sh-NT CRC cells-derived EVs, but the uptake of EVs by sh-SNAP23 cells promotes tumor growth. Tumor cells might not only selectively secrete but also uptake of various types of EVs to keep alive. Let-7 family has been recently found to control both pro- and anti-inflammatory responses in macrophages by regulating the accumulation of the key metabolite genes [45, 46]. It suggests that we could focus on the roles of extracellular let-7 in cellular metabolism and energy in immune cells or other tumor-associated cells. And the mechanisms of let-7a/SNAP23 pathway in tumor cells need to be more specific.

## Conclusions

In summary, our current study reveals that let-7a could regulate EV secretion and mitochondrial OXPHOS by targeting SNAP23. This finding elucidates a new molecular mechanism underlying the crosstalk between EV secretion and cell metabolic switch to promote tumor progression, in which let-7a/SNAP23 may provide novel approaches for CRC therapy.

## Supplementary Information

**Additional file 1: Table S1.** Primer sequences and target sequences. **Table S2.** Comparison of clinical pathologic characteristics of let-7a in serum EVs from CRC patient. **Table S3.** Comparison of clinical pathologic characteristics of CRC patient tissue with SNAP23 expression.

**Additional file 2: Figure S1.** let-7a and SNAP23 expression in cell lines. qPCR of let-7a (a) and western blotting of SNAP23 (b) in CRC cell lines and FHC.

**Additional file 3: Figure S2.** qPCR of intra- and extracellular miRNAs in CRC sh-SNAP23 cells. (a) Western blot of indicated proteins in SW620 and SW480 cells transfected with different shRNA-SNAP23 or shRNA-NT, with β-Actin as the loading control. According the effect of RNA interference, we used the sh-SNAP23–3 sequence in this study to further investigation. (b, c) qPCR of intra- and extracellular miR-10a-5p and miR-16-5p (unrelated to let-7a) in SW620 sh-SNAP23 cells. Data represent the mean ± SEM of at least three independent experiments.

**Additional file 4: Figure S3.** Let-7a/SNAP23 suppresses the growth of CRC cells. (a, c) Cell migration was determined by wound healing assay in SW620 cells overexpressing vector and SNAP23 treated with ctrl or let-7a mimic. Original magnification: 100×. (b, d) Cell migration was shown in sh-NT and sh-SNAP23 SW480 cells treated with ctrl or let-7a inhibitor. Original magnification: 100×. (e) The proliferation of sh-NT and sh-SNAP23 SW620 transfected with ctrl or let-7a mimic. (f) Cell migration was determined by wound healing assay. (g) Cell invasion was confirmed using transwell assay. Scale bar, 200 μM. Original magnification: 200×. Data represent the mean ± SEM of at least three independent experiments. *p < 0.05, **p < 0.01 and ***p < 0.001.

**Additional file 5: Figure S4.** Let-7a suppresses glycolysis in CRC cells. Extracellular acidification rate (ECAR) was assayed using the Seahorse analyzer in SW620 transfected with let-7a mimic (a) or SW480 transfected with let-7a inhibitor (b) with the treatment of glucose, oligomycin and 2-deoxy-glucose (2-DG). (c) PKM2 and p-PKM2 expression was determined in CRC cells after the transfection of let-7a mimic or inhibitor and sh-SNAP23 or overexpressing SNAP23. Data represent the mean ± SEM of at least three independent experiments.

## Data Availability

The dataset supporting the conclusions of this article is included within the article.

## Abbreviations

EVs: Extracellular vesicles
miRNA: microRNA
SNAP23: Synaptosome-associated protein 23
CRC: Colorectal cancer
FCS: Fetal calf serum
HC: heathy controls
UC: Ultracentrifugation
OXPHOS: oxidative phosphorylation
