# Supplementary material for: Let-7a regulates EV secretion and mitochondrial oxidative phosphorylation by targeting SNAP23 in colorectal cancer
Source: J Exp Clin Cancer Res. 2021 Jan 14;40:31. doi: 10.1186/s13046-020-01813-6 (PMC7807815; doi:10.1186/s13046-020-01813-6)
Supplement: Supplementary file 1 — Additional file 1: Table S1. Primer sequences and target sequences. Table S2. Comparison of clinical pathologic characteristics of let-7a in serum EVs from CRC patient. Table S3. Comparison of clinical pathologic characteristics of CRC patient tissue with SNAP23 expression. [file 13046_2020_1813_MOESM1_ESM.docx]

**Additional file 1**

**Additional file 1: Table S1 Primer sequences and target sequence.**

| **Name** | **Forward** |
| --- | --- |
| **18S forward** | CGGCTACCACATCCAAGGAA |
| **18S reverse** | GCTGGAATTACCGCGGCT |
| **SNAP23 forward** | CTGCCCATGTAATAGAACAAAGAAC |
| **SNAP23 reverse** | CGGGCCTGGCTGTTTAGATA |
| **TFAM forward** | CGCTCCCCCTTCAGTTTTGT |
| **TFAM reverse** | CCAACGCTGGGCAATTCTTC |
| **CREB1 forward** | ACCAAGTTGTTGTTCAAGGTACT |
| **CREB1 reverse** | ACATGTTACCATCTTCAAACTGACG |
| **Lin28a forward** | CCAGTGGATGTCTTTGTGCACC |
| **Lin28a reverse** | GTGACACGGATGGATTCCAGAC |
| **Lin28b forward** | CCTGTTTAGGAAGTGAAAGAAGAC |
| **Lin28b reverse** | CCTGTTTAGGAAGTGAAAGAAGAC |
| **VAMP3 forward** | GCTCTCTGAGTTAGACGACCGT |
| **VAMP3 reverse** | CCAGAACAGTAATCCCGATTGCC |
| **Rab35 forward** | CAGCCCATCTTACTGCAAGCAG |
| **Rab35 reverse** | GCTGACAACCTGTCGGAGAGAA |
| **U6 forward** | GCTCGCTTCGGCAGCACATATAC |
| **U6 reverse** | GTCGTATCGCTCGCTTCGGCAGCACATATACCAGTGCAGGGTCCGAGGTATTCGCACTGGATACGACAAAATATGG |
| **Universal-reverse** | AGTGCAGGGTCCGAGGTATT |
| **Let-7a forward** | GCGCGTGAGGTAGTAGGTTGT |
| **Let-7a reverse** | GTCGTATCCAGTGCAGGGTCCGAGGTATTCGCACTGGATACGACAACTAT |
| **sh-SNAP23-1 forward** | GCTTGGACCAAATAAATAA |
| **sh-SNAP23-1 reverse** | GCTTGGACCAAATAAATAA |
| **sh-SNAP23-2 reverse** | GCAGTATCCTGGGAAATCT |
| **sh-SNAP23-2 forward** | GCAGTATCCTGGGAAATCT |
| **sh-SNAP23-3 reverse** | GCCACAACGAAGATCTAGT |
| **sh-SNAP23-3 forward** | GCCACAACGAAGATCTAGT |
| **SNAP23 vector reverse** | ggatccTTACTTGTCGTCATCGTCTTTGTAGTCGCTGTCAATGAGTTTCTTTGCT |
| **SNAP23 vector forward** | gaattcGCCACCATGGATAATCTGTCATCAGAAGAA |

**Additional file 1: Table S2 Comparison of clinical pathologic characteristics of let-7a in serum EVs from CRC patient.**

| Variable | | n | Low | High | Pearson x | p Value |  |
| --- | --- | --- | --- | --- | --- | --- | --- |
| Total | | 31 | 15 | 16 |  |  |  |
|  | Gender | | | | | | |
| Male  Female | | 18  13 | 10 55.6%  5 38.5% | 8 44.4%  8 61.5% | 0.883 | 0.347 |  |
|  | Age | | | | | | |
| <65  >=65 | | 7  24 | 5 71.4%  10 41.7% | 2 28.6%  14 58.3% | 1.922 | 0.166 |  |
|  | Distant metastasis | | | | | | |
| No  Yes | | 25  6 | 12 48.0%  3 50.0% | 13 52.0%  3 50.0% | 0.008 | 0.930 |  |
|  | AJCC stage | | | | | | |
| 1-2  3-4 | | 9  22 | 6 66.7%  9 40.9% | 3 33.3%  13 59.1% | 1.697 | 0.193 |  |

**Additional file 1: Table S3 Comparison of clinical pathologic characteristics of CRC patient tissue with SNAP23 expression.**

| Variable | | n | Weak | Moderate | Strong | Pearson x | p Value |
| --- | --- | --- | --- | --- | --- | --- | --- |
| Total | | 31 | 7 | 13 | 11 |  |  |
|  | Gender | | | | | | |
| Male  Female | | 18  13 | 4 22.2%  3 23.1% | 6 33.3%  7 53.8% | 8 44.4%  3 23.1% | 1.731 | 0.421 |
|  | Age | | | | | | |
| <65  >=65 | | 7  24 | 3 42.9%  4 16.7% | 2 28.6%  11 45.8% | 2 28.6%  6 37.5% | 2.153 | 0.341 |
|  | Distant metastasis | | | | | | |
| No  Yes | | 25  6 | 6 24.0%  1 16.7% | 10 40.0%  3 50.0% | 9 36.0%  2 33.3% | 0.240 | 0.887 |
|  | AJCC stage | | | | | | |
| 1-2  3-4 | | 9  22 | 4 44.4%  3 13.6% | 2 22.2%  11 50.0% | 3 33.3%  8 36.4% | 3.876 | 0.144 |
