# Supplementary figures and images for: Let-7a regulates EV secretion and mitochondrial oxidative phosphorylation by targeting SNAP23 in colorectal cancer
Source: J Exp Clin Cancer Res. 2021 Jan 14;40:31. doi: 10.1186/s13046-020-01813-6 (PMC7807815; doi:10.1186/s13046-020-01813-6)

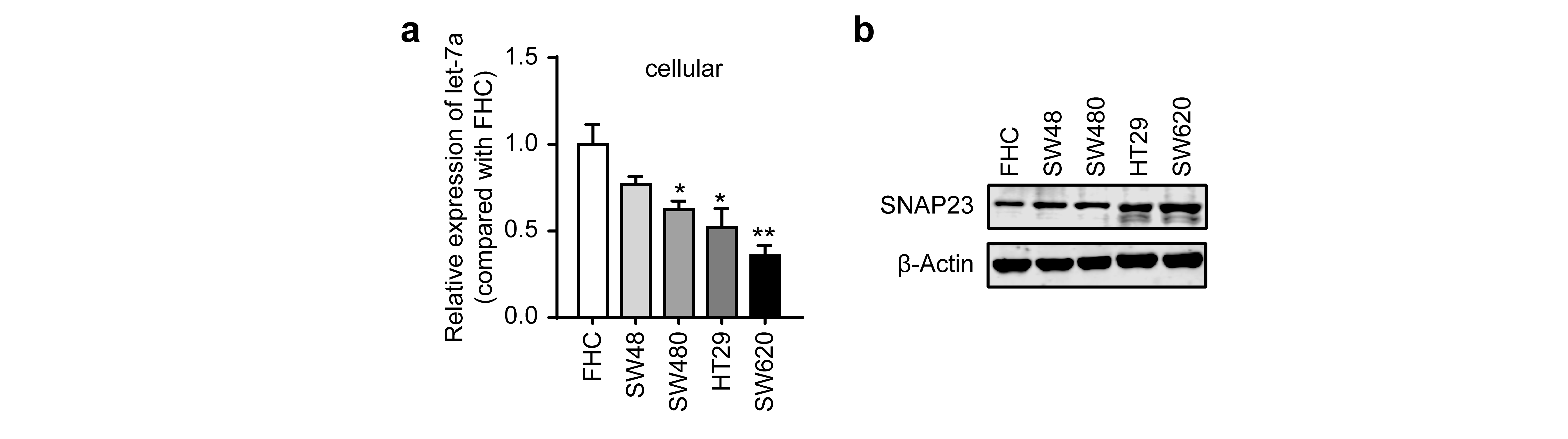

Supplement: Supplementary file 2 — Additional file 2: Figure S1. let-7a and SNAP23 expression in cell lines. qPCR of let-7a (a) and western blotting of SNAP23 (b) in CRC cell lines and FHC. [file 13046_2020_1813_MOESM2_ESM.tif]

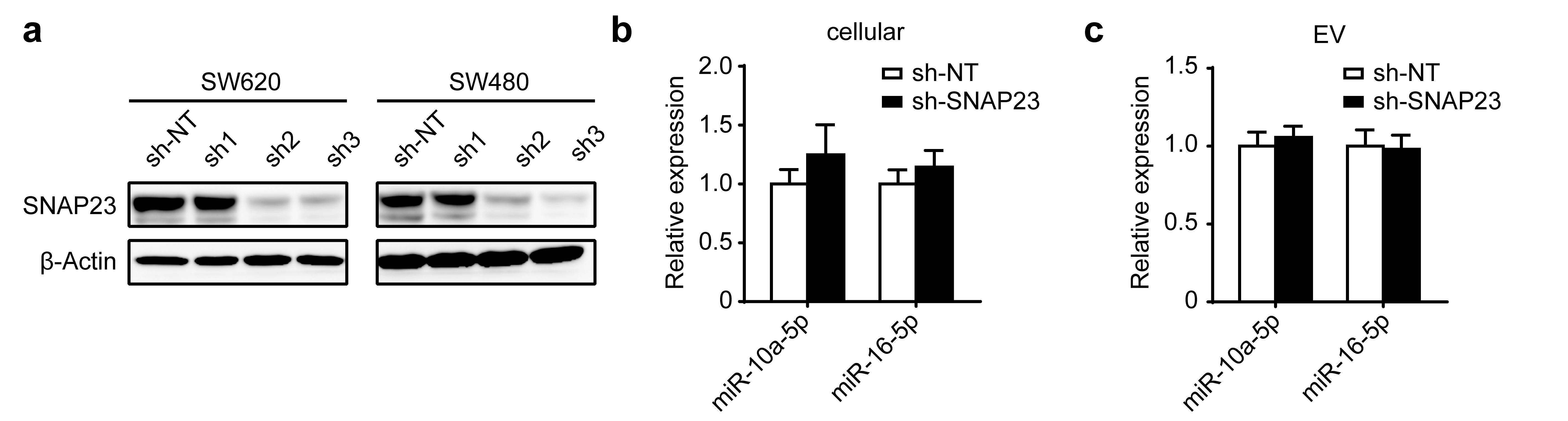

Supplement: Supplementary file 3 — Additional file 3: Figure S2. qPCR of intra- and extracellular miRNAs in CRC sh-SNAP23 cells. (a) Western blot of indicated proteins in SW620 and SW480 cells transfected with different shRNA-SNAP23 or shRNA-NT, with β-Actin as the loading control. According the effect of RNA interference, we used the sh-SNAP23–3 sequence in this study to further investigation. (b, c) qPCR of intra- and extracellular miR-10a-5p and miR-16-5p (unrelated to let-7a) in SW620 sh-SNAP23 cells. Data represent the mean ± SEM of at least three independent experiments. [file 13046_2020_1813_MOESM3_ESM.tif]

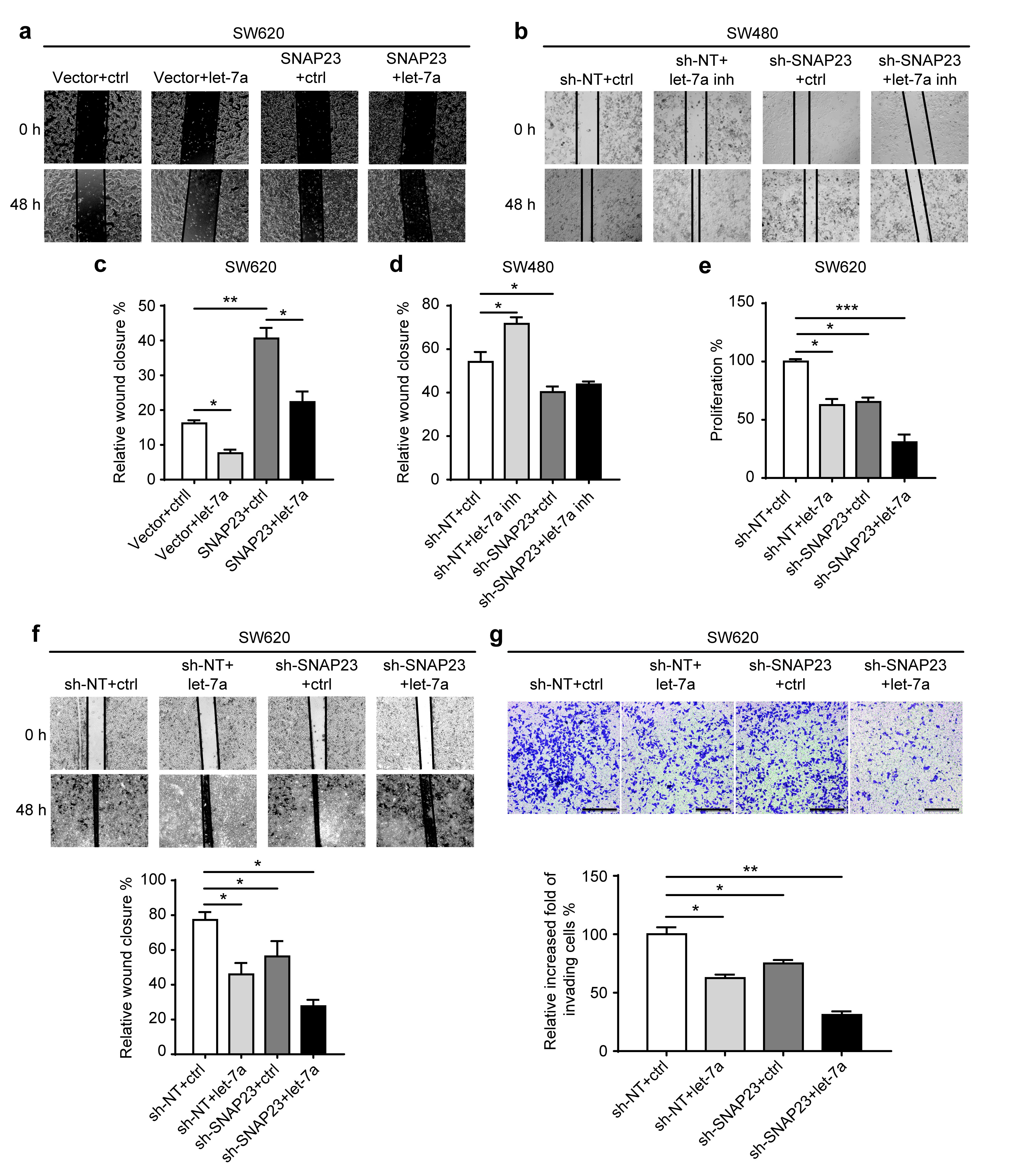

Supplement: Supplementary file 4 — Additional file 4: Figure S3. Let-7a/SNAP23 suppresses the growth of CRC cells. (a, c) Cell migration was determined by wound healing assay in SW620 cells overexpressing vector and SNAP23 treated with ctrl or let-7a mimic. Original magnification: 100×. (b, d) Cell migration was shown in sh-NT and sh-SNAP23 SW480 cells treated with ctrl or let-7a inhibitor. Original magnification: 100×. (e) The proliferation of sh-NT and sh-SNAP23 SW620 transfected with ctrl or let-7a mimic. (f) Cell migration was determined by wound healing assay. (g) Cell invasion was confirmed using transwell assay. Scale bar, 200 μM. Original magnification: 200×. Data represent the mean ± SEM of at least three independent experiments. *p < 0.05, **p < 0.01 and ***p < 0.001. [file 13046_2020_1813_MOESM4_ESM.tif]

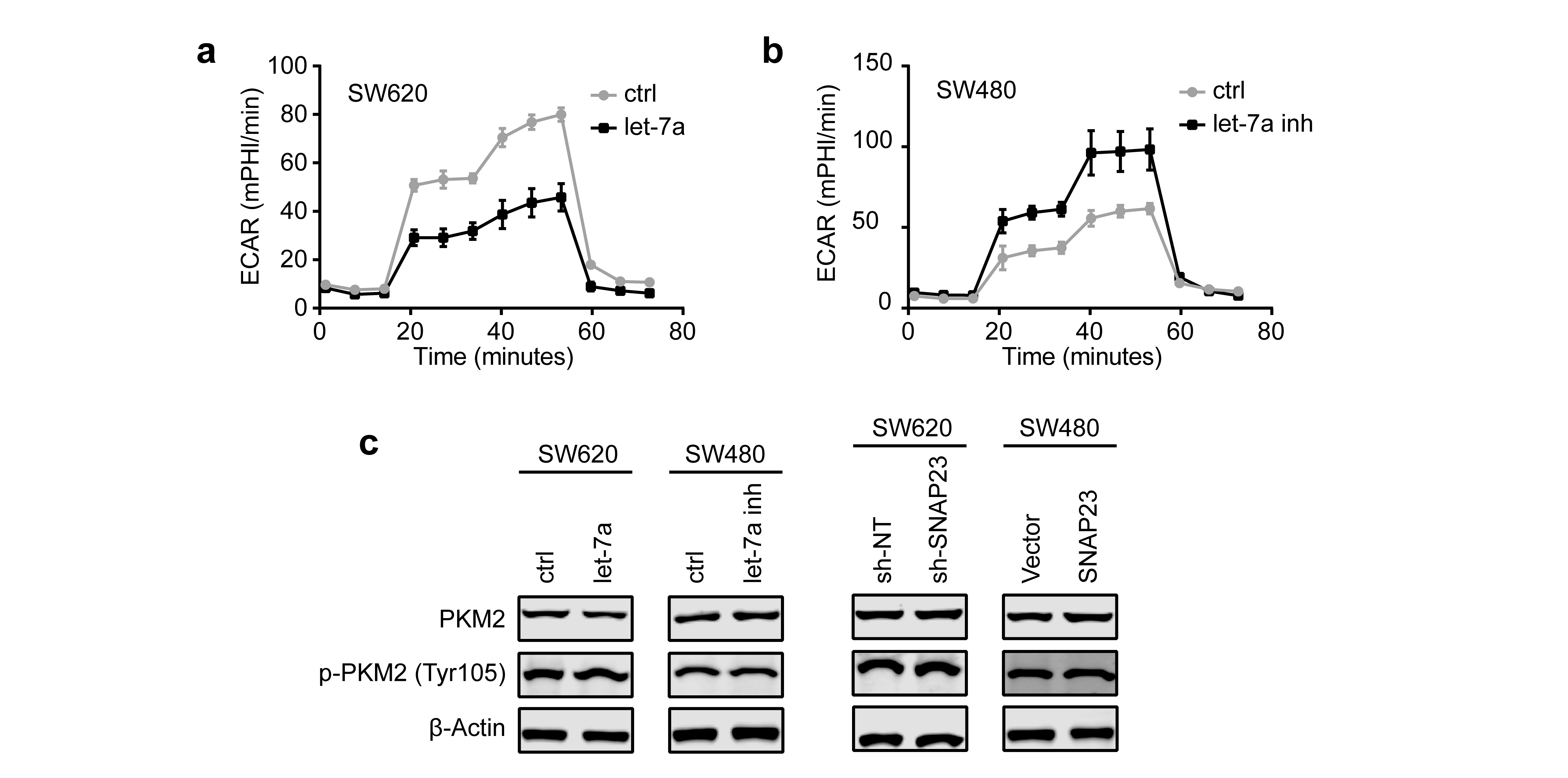

Supplement: Supplementary file 5 — Additional file 5: Figure S4. Let-7a suppresses glycolysis in CRC cells. Extracellular acidification rate (ECAR) was assayed using the Seahorse analyzer in SW620 transfected with let-7a mimic (a) or SW480 transfected with let-7a inhibitor (b) with the treatment of glucose, oligomycin and 2-deoxy-glucose (2-DG). (c) PKM2 and p-PKM2 expression was determined in CRC cells after the transfection of let-7a mimic or inhibitor and sh-SNAP23 or overexpressing SNAP23. Data represent the mean ± SEM of at least three independent experiments. [file 13046_2020_1813_MOESM5_ESM.tif]
